# Evaluation Of Perceived Stress Among Anesthesia Technicians

**DOI:** 10.1192/j.eurpsy.2026.12098

**Published:** 2026-07-31

**Authors:** G. Mohamed Anis, S. Imen, H. Aicha, F. Afef, H. Asma, M. Mohamed Larbi, H. Mounira, H. J. Kaouthar

**Affiliations:** 1 Occupational Medicine; 2 Rheumatology Department, University Hospital Hedi Chaker; 3 Physiology and Functional Explorations, University Hospital Habib Bourguiba, Sfax, Tunisia

## Abstract

**Introduction:**

Work-related stress in the healthcare sector is a major challenge due to its impact on the quality of care and the well-being of healthcare workers. Among the most exposed units, operating rooms stand out for their highly demanding environment, characterized by urgency, technical complexity, and the vital responsibility of the procedures performed.

**Objectives:**

The aim of this study was to assess perceived stress among anesthetic technicians (AT) and evaluate its associated factors.

**Methods:**

We conducted a cross-sectional study among AT in two University Hospitals in Sfax, Tunisia, between January and October 2024. Sociodemographic and professional data were collected. Physical exertion was evaluated on a scale of 1 to 10. Perceived stress was evaluated using the Perceived-Stress-Scale-10 (PSS-10).

**Results:**

Our population consisted of 77 AT with a mean age of 47.32±7.74 years. Five participants (6.5%) were males. The median Body Mass Index (BMI) of our population was 25.71 IQR [23.87; 28.94]. Medical history was noted in 93.5% of the population. Psychiatric and neurological diseases were found in 15.6% and 9.1% of the population respectively. Physical and Leisure activities were found in 13% and 24.7% of the population respectively. The median job tenure was 7 years IQR [3; 13]. Fourteen participants (18.2%) worked in Pediatric Surgery Unit (PSU). The median of the physical exertion was 7 IQR [5; 8]. The mean of the PSS-10 score was 18.5±6.5. Moderate and high scores were found in 66.2% and 13% of the population, respectively. Multivariate analysis showed that BMI (β=0.213; p=0.049), physical exertion (β=0.429; p<0.001), working in PSU (β=0.271; p=0.019) were significantly associated with a higher PSS-10 score. Job satisfaction was associated with a lower PSS-10 score (β=-0.233; p=0.032).

**Conclusions:**

Our results showed that higher stress was observed in AT working with pediatric patients whereas job satisfaction was associated with lower stress score. These results call for the implementation of adequate support strategies and pave the way for future research on stress prevention.

**Disclosure of Interest:**

None Declared

